# The Human Bone Marrow Is Host to the DNAs of Several Viruses

**DOI:** 10.3389/fcimb.2021.657245

**Published:** 2021-04-22

**Authors:** Mari Toppinen, Antti Sajantila, Diogo Pratas, Klaus Hedman, Maria F. Perdomo

**Affiliations:** ^1^ Department of Virology, University of Helsinki and Helsinki University Hospital, Helsinki, Finland; ^2^ Department of Forensic Medicine, University of Helsinki, Helsinki, Finland; ^3^ Forensic Medicine Unit, Finnish Institute for Health and Welfare, Helsinki, Finland; ^4^ Department of Electronics, Telecommunications and Informatics, University of Aveiro, Aveiro, Portugal; ^5^ Institute of Electronics and Informatics Engineering of Aveiro, University of Aveiro, Aveiro, Portugal

**Keywords:** human bone marrow, DNA viruses, human papillomavirus 31, transplantation, hematopoietic disorders, virome

## Abstract

The long-term impact of viruses residing in the human bone marrow (BM) remains unexplored. However, chronic inflammatory processes driven by single or multiple viruses could significantly alter hematopoiesis and immune function. We performed a systematic analysis of the DNAs of 38 viruses in the BM. We detected, by quantitative PCRs and next-generation sequencing, viral DNA in 88.9% of the samples, up to five viruses in one individual. Included were, among others, several herpesviruses, hepatitis B virus, Merkel cell polyomavirus and, unprecedentedly, human papillomavirus 31. Given the reactivation and/or oncogenic potential of these viruses, their repercussion on hematopoietic and malignant disorders calls for careful examination. Furthermore, the implications of persistent infections on the engraftment, regenerative capacity, and outcomes of bone marrow transplantation deserve in-depth evaluation.

## Introduction

Hematopoiesis occurs in the bone marrow (BM) as a result of the crosstalk between, among others, mesenchymal and hematopoietic stem cells. Their interaction occurs in niches in which complex networks are built upon concerted cell-to-cell architectures. These compartments have been delineated in animal models at single-cell resolution (Tikhonova et al., 2019; Baccin et al., 2020), revealing a large diversity in the cellular components of arteriolar, endosteal, and sinusoidal niches. This heterogeneity gives rise to unique signaling patterns accounting for the multiple effector functions of this organ.

Alterations in these microenvironments, e.g. by viral infections, may result in multiple pathological conditions. Indeed, persistent viruses can perturb the homeostasis, either by cytotoxic damage of the niche´s structural integrity, transformation of the signaling repertoire, or interference with the cellular trafficking (Alexaki and Wigdahl, 2008; Pascutti et al., 2016). However, while the pathogenic effects of viruses in the BM are primarily known in the context of acute infection (Scadden et al., 1989; Rosenfeld and Young, 1991; Luppi et al., 2000) the full spectrum of viruses here persisting and the long-term repercussions on the marrow’s physiology and composition remain poorly understood.

Moreover, while most studies have focused on single viruses (Baranski et al., 1988; Cantin et al., 1994; Cassinotti et al., 1997; Eis-Hübinger et al., 2001; Heegaard et al., 2002; Khaiboullina et al., 2004; Lundqvist et al., 2005; Manning et al., 2007; Rollín et al., 2007; Corcioli et al., 2008; Tan et al., 2009; Garcia et al., 2011; Matsushita et al., 2013; Pambrun et al., 2014), a comprehensive analysis of the different types concomitantly persisting in the BM of an individual is lacking. This is however important, as their chronic effects, either concerted or combined, may contribute to BM dysfunction.

In the present study, we systematically investigated the genomic prevalence and copy numbers of altogether 38 clinically significant DNA viruses in the BM of 27 recently deceased individuals. Included were members of the *Parvoviridae, Herpesviridae, Polyomaviridae, Hepadnaviridae, Anelloviridae*, and *Papillomaviridae* families. To the best of our knowledge, this is the most comprehensive assessment of viral DNAs present in the BM to date.

## Methods

### Study Cohort

**Figure 1:**
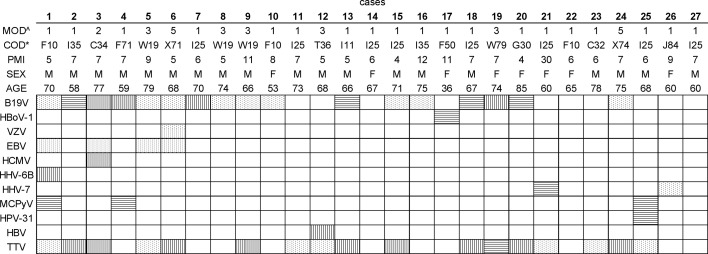
Viral DNA prevalences in bone marrow per study subject. The viral findings are presented as dots (NGS+, qPCR+), horizontal lines (NGS+, qPCR-), vertical lines (NGS-, qPCR+), and white (NGS-, qPCR-). ^The manner of death (MOD) is presented as 1=disease, 2=occupational disease, 3=injury, 5=suicide. *The cause of death (COD) is given according to the WHO ICD10 classification (https://icd.who.int/browse10/2019/en). Age is given in years. PMI, Post-mortem interval (given in days); B19V, parvovirus B19; HBoV-1, human bocavirus 1; VZV, varicella-zoster virus; EBV, Epstein-Barr virus; HCMV, cytomegalovirus; HHV-6B, human herpesvirus 6B; HHV-7, human herpesvirus 7; MCPyV, Merkel cell polyomavirus; HPV-31, human papillomavirus type 31; HBV, hepatitis B virus; TTV, torque teno virus.

The study cohort consisted of 27 deceased individuals of Finnish origin. The age range of the study subjects was 36 to 85 years (mean 68) and the postmortem interval on average 8 days (**Figure 1**). None of the individuals had records of the use of immunosuppressants, neither of virus-related cancers or ongoing viral infections at the time of death, except for one (case number 6) with a history of facial shingles a few weeks earlier. The causes and manners of deaths are presented in **Figure 1**. The study was reviewed by the Ethics Committee of Helsinki and Uusimaa Hospital District (approval 164/13/03/00/2014).

### Sample Processing

The BM was collected from the diaphysis of femoral bone and the DNA extracted with QIAamp DNA Mini Kit (Qiagen) as per manufacturer protocol. The quality and integrity of total DNA in each sample was confirmed with a Genomic DNA Reagent Kit in a LabChip GX Instrument.

To avoid contamination, strict precautions were taken throughout the experiments including the use of single-use disposable plastics and filter tips, the handling in hoods dedicated exclusively to nucleic acid work, and the use of negative controls through all steps. The reaction mixes, DNA extracts, plasmid controls, and amplification reactions, were each handled in separate rooms.

### Virus Analysis

The viral DNAs were analyzed by targeted enrichment followed by sequencing in NovaSeq 6000 (Illumina), as well as by quantitative PCRs.

For the enrichment, a customized panel of biotinylated RNA oligonucleotides (Arbor Biosciences) was used. The viruses targeted were parvovirus B19 (B19V), human bocaviruses 1-4 (HBoV1-4), cutavirus, human papillomavirus (HPV) types 2, 6, 11, 16, 18, 21, and 45, human polyomaviruses (HPyV) 1-13, simian virus 40, human herpesviruses (HHV) 1-8, hepatitis B virus (HBV), torque teno virus (TTV) and variola virus (minor and major). A complete list of the reference strains used for the design of the RNA oligonucleotides, as well as the details of the NGS methods, are presented in (Toppinen et al., 2020). In brief, the sequencing libraries were prepared from sonicated total DNA (10-1000 ng) using the KAPA HyperPlus library preparation kit (Roche). The viral genomes were enriched with MyBaits v4 kit (Arbor Biosciences) using two consecutive rounds of hybridization on individual samples, following the manufacturer recommendations for low input DNA. The viral genomic sequences were reconstructed after removal of PCR duplicates, using a customized bioinformatics pipeline, TRACESPipe (Pratas et al., 2020). The adapter sequences were removed using Trimmomatic (Bolger et al., 2014), by explicitly removing content from an adapters’ list having a maximum mismatch that allowed a full match of 2. The palindrome and simple clip threshold were set at 30 and 10, respectively. The minimum quality score required to keep a base at the beginning and the end was fixed to 3. Low-quality data was filtered using a sliding window of 4 with an average quality of 15 and low complexity regions were flagged with GTO (Almeida et al., 2020). Reads shorter than 25 bases were discarded.

The consensus, as well as single sequences (when in low coverage), were confirmed by BLAST (NCBI).

To confirm the NGS findings, validated in-house quantitative PCRs were used for the detection of B19V (Toppinen et al., 2015), Merkel cell polyomavirus (MCPyV) (Goh et al., 2009), TTV (Toppinen et al., 2020), and the nine HHVs (Pyöriä et al., 2020). HBV and HPV-31 were tested with commercial kits (Hepatitis B Virus PCR Kit, GeneProof, Genesig; and Human papillomavirus 31 Standard kit, PrimerDesign, respectively) according to the manufacturer protocol. The human single-copy gene RNase P qPCR was utilized for normalization of the viral DNA copies to the cell number (Toppinen et al., 2015). See **Supplementary Table 1** for further information on the qPCRs used.

## Results

We conducted a systematic analysis of viral DNAs present in the bone marrow by two complementary approaches, NGS and qPCR.

**Figure 2:**
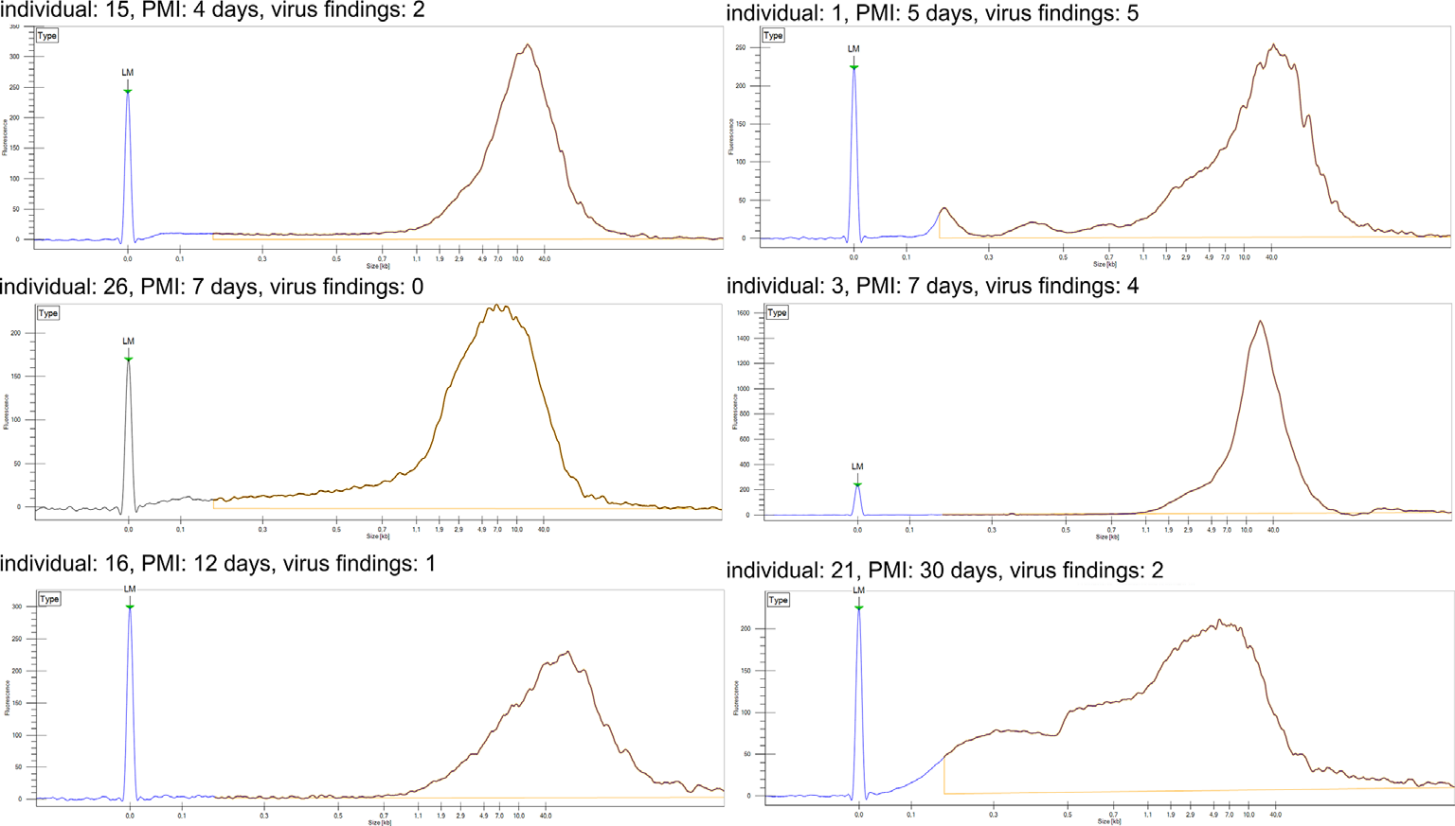
DNA fragment analysis of total DNA. Fragment length distribution of genomic DNA for six representative samples as established with LabChip GX. Individual number refers to **Figure 1**. LM, lower marker; PMI, post-mortem interval.

We first analyzed the integrity of the total genomic DNA of each sample to control for bias in the prevalences and loads detected by qPCR. Based on the size distribution, the total DNA was of high quality and non-fragmented, except for one sample, taken 30 days post-mortem, exhibiting a wider range of fragment sizes (**Figure 2**).

We detected viral DNA in 88.9% of the samples, comprising altogether 11 distinct virus types (**Table 1**). The most prevalent genomes were of B19V and TTV each found in 62.9% of the individuals, followed by Epstein-Barr virus (EBV) in 14.8%, MCPyV in 11.1%, and human herpesvirus 7 (HHV-7) in 7.4%. Other viral genomes, identified in single cases, were of varicella-zoster virus (VZV), human cytomegalovirus (HCMV), human herpesvirus 6B (HHV-6B), HBoV-1, HPV-31, and HBV.

**Table 1:** DNA virus findings from human bone marrow.

Family	Virus	Genoprevalence	NGS+ cases	qPCR+ cases	breadth coverage	viral load/1E6 cells
*Parvoviridae*	B19V	62.9%	13	13	2.5-97.2%	7.9E3
	HBoV-1	3.7%	1	0	4.3%	–
*Herpesviridae*	VZV	3.7%	1	1	2.9%	2.5E1
	EBV	14.8%	4	4	0.9-8.2%	9.1E1
	HCMV	3.7%	0	1	–	1.0E2
	HHV-6B	3.7%	0	1	–	3.8E1
	HHV-7	7.4%	2	1	6.7-7.8%	3.4E2
*Polyomaviridae*	MCPyV	11.1%	3	0	2.2-17.9%	–
*Papillomaviridae*	HPV-31	3.7%	1	0	11.9%	–
*Hepadnaviridae*	HBV	3.7%	0	1	–	1.2E3
*Anelloviridae*	TTV	62.9%	8	16	3.7-23.6%	2.8E5

B19V, parvovirus B19; HBoV-1, human bocavirus 1; VZV, varicella-zoster virus; EBV, Epstein-Barr virus; HCMV, cytomegalovirus; HHV-6B, human herpesvirus 6B; HHV-7, human herpesvirus 7; MCPyV, Merkel cell polyomavirus; HPV-31, human papillomavirus type 31; HBV, hepatitis B virus; TTV, torque teno virus.

Breadth coverage is shown as range of values or, in the case of a single sample, the breadth coverage of that sample. The viral loads are normalized per million cells and correspond to either median value or, in the case of a single positive sample, the viral load of that sample.

On average, we identified two virus types per individual, the most common co-detections being of B19V and TTV, in 44.4% of the BMs (**Figure 1**). A third of these double-positive marrows were also positive for EBV (i.e. 14.8% of all cases). The maximum number of viruses per individual was five, corresponding to a single case in which B19V, EBV, HHV-6B, MCPyV, and TTV were found.

When normalized to copies per million cells, the highest quantities were of B19V and TTV with a median of 7.9E3 and 2.8E5, respectively, to be followed by EBV with 9.1E1 copies. VZV, HCMV, HHV-6B, HHV-7 and HBV had respectively 2.5E1, 1.0E2, 3.8E1, 3.4E2 and 1.2E3 copies. In general, the quantities of most viral DNAs were very low, speaking against active replication.

**Figure 3:**
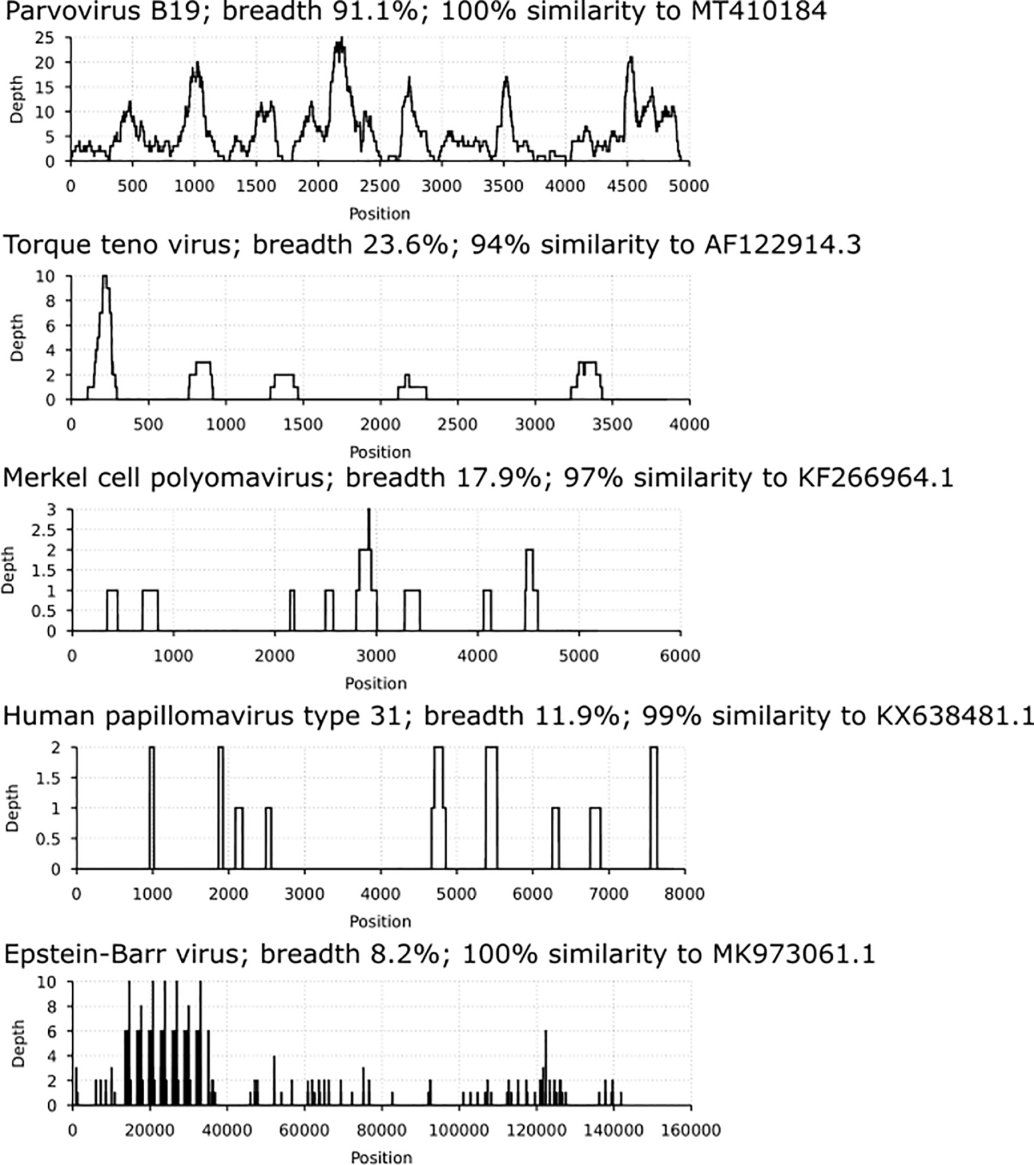
Coverage profiles of reconstructed viral DNA sequences. In the *x axis* is the breadth coverage (= reads covering the noted viral reference) and in the *y axis* the depth (number of reads covering a specific nucleotide/area). Represented are parvovirus B19, torque teno virus, Merkel cell polyomavirus, human papillomavirus type 31 and Epstein-Barr virus. Each profile is specified by the virus name, breadth coverage and the genomic sequence similarity to its closest match by NCBI-BLAST.

Via in-solution hybridization, we captured and reconstructed 11 viral genomic sequences, with a minimum breadth coverage of 15% (**Figure 3**). Highest qualities were obtained for B19V (n=7, average breath coverage 88.2%; Genbank accession numbers MT988397-MT9888403), MCPyV (n=1, breath 17.9%; **Supplementary Table 2**), and TTV (n=3, average breath 19.8%; **Supplementary Table 3**). Remarkably, we found in one individual 14 unique reads (total length 936 nucleotides, **Supplementary Table 4**) matching to human papillomaviruses, with the highest similarity to HPV-31.

Moreover, albeit in low coverage, we manually and visually confirmed by BLAST each of the unique sequences (mean length 100 nucleotides) specific for the following viruses: VZV (1 individual, 103 reads), EBV (4 individuals, altogether 342 reads), HHV-7 (2 individuals, altogether 76 reads), MCPyV (2 additional findings, a total of 5 reads), TTV (6 additional findings, altogether 22 reads), B19V (5 additional findings, altogether 66 reads), and HBoV-1 (1 individual, 2 reads).

Because of our cohort’s advanced age and the sampling location, a higher proportion of mesenchymal cells are to be expected. We compared the sequences of the viruses found in the BM with those detected in the same individuals´ external surface of the femoral bone (Toppinen et al., 2020). We found a match in one or more individuals for EBV, HCMV, HHV-6B, MCPyV, B19V, TTV, and HBV. Having the reconstructed sequences of B19V the highest quality, we analyzed the nucleotide similarities between bone and BM and found they were nearly identical (with a maximum of four single nucleotide polymorphisms; **Supplementary Table 5**).

The reconstructed consensus sequences of B19V have been deposited in GenBank with accession numbers: MT988397-MT988403.

## Discussion

A comprehensive analysis of the virus types residing in the human bone marrow is lacking. Yet, this knowledge is essential for assessment of the impact of viral DNA persistence on the marrow’s integrity and on the emergence of many pathological conditions. Since the BM homeostasis is founded on many precise regulatory networks, any alterations resulting from single or cumulative effects of multiple viruses can foreseeably have profound effects on its output and function.

We evaluated the prevalences of viral DNAs residing in the bone marrow and found 11 different virus types, in copy numbers compatible with latency.

Remarkably, among the viral genomes identified, we found HPV-31 in a male individual aged 68, who died of atherosclerotic heart disease. Since infections by this oncovirus are normally self-limited and restricted to epithelial surfaces, the detection of its DNA in BM is perplexing. Other HPV types have been reported in BM by Payne et al. (Payne et al., 1995; Payne et al., 1996), albeit in patients with multiple myeloma and monoclonal gammopathy of unknown significance. Our discovery in BM is supported by a parallel retrieval of the full-length genome of HPV-31 in the femoral bone of another individual (Toppinen et al., 2020). Unfortunately, we were unable to match the detection of this virus type in the bone and BMs of the subjects, possibly due to the very low copy numbers. Since neither individual had an HPV related clinical history, nor compatible pathological findings at the post-mortem evaluation, our discovery points to the possibility of bone and BM as novel sites of HPV-31 persistence and raise critical questions on its clinical significance (Flynn et al., 2010).

Another interesting finding was that of the genome of MCPyV, likewise of mucocutaneous tropism and oncogenic potential. We detected it in three individuals, two of whom were also positive in the bone. MCPyV DNA in BM was reported by Matsushita et al. (Matsushita et al., 2013), who found a similar prevalence (17%) in Japanese cadavers, but not by Dang et al. (2011) who surveyed also KI and WU polyomaviruses in 13 patients with different medical conditions.

Importantly, advanced stages of malignant transformation by both papillomaviruses and Merkel cell polyomavirus can present with manifestations in bone (Kamijo et al., 2002; Ley et al., 2014; Lee et al., 2018; Lewis et al., 2020). While normally considered metastatic, the contribution of the viruses already residing in these tissues should be assessed.

Our study is limited by the lack of distinction of the specific host-cell types. To date, the cellular reservoirs of most viruses in the BM are actually unknown, although *in vitro* a wide range of constituent and memory cells have been shown permissive for one or more (Chiara et al., 1996; Movassagh et al., 1996; Bahner et al., 1997; Babcock et al., 1998; Okamoto et al., 2000; Khaiboullina et al., 2004; Luppi et al., 2005; Sundin et al., 2006; Wu et al., 2006; Rollín et al., 2007; Smirnov et al., 2007; Alexaki and Wigdahl, 2008; Rong et al., 2008; Sundin et al., 2008; Ma et al., 2011; Bua et al., 2016; Furuta et al., 2017; Pyöriä et al., 2017).

When comparing the viral genome findings in the BM to those of the adjacent femoral bone, we found a match for EBV, HCMV, HHV-6B, MCPyV, B19V, TTV, and HBV in one or more individuals. This raises the possibility of infection taking place already at the precursor stage, whereafter the virus could be carried through the mature cell populations constituents of bone. Previously, stem cells have been proposed as renewable sources of B19V (Sundin et al., 2008), HCV (Ivantes et al., 2004), HIV (Alexaki and Wigdahl, 2008), and JCPyV (Chiara et al., 1996), thereby contributing to viral spread and disease progression. Taken together, our results call for further study on the implications of the resident viral genomes to the bone’s structure and metabolism, a topic until now unexplored.

Given the critical roles of BM in the support and regulation of hematopoiesis, lineage-specific differentiation, as well as homing and survival of memory cells (Visnjic et al., 2004; Bowers et al., 2015), our findings call for careful consideration of the clinical implications of viral persistence. Indeed, since many of the viruses detected can reactivate, special attention should be paid to their association with the development of lymphoproliferative and/or malignant disorders (Bartenhagen et al., 2017). Furthermore, the burden of persistent infections on the engraftment, regenerative capacity, and outcomes (Ivantes et al., 2004; Nombela-Arrieta and Isringhausen, 2017) of BM transplantation deserve in-depth evaluation.

In summary, our findings provide evidence of a wide spectrum of viral DNAs present in the human BM. In-depth studies are essential to determine their pathogenic potential and clinical significance.

## Data Availability Statement

The datasets presented in this study can be found in online repositories. The names of the repository/repositories and accession number(s) can be found in the article/**Supplementary Material**.

## Ethics Statement

The studies involving human participants were reviewed and approved by Ethics Committee of Helsinki and Uusimaa Hospital District (approval 164/13/03/00/2014). Written informed consent for participation was not required for this study in accordance with the national legislation and the institutional requirements.

## Author Contributions

MT, AS, KH, and MP designed the study. MT performed the experiments. MT, DP, and MP analyzed the data. All authors contributed to the article and approved the submitted version.

## Funding

This work was supported by grants from the Finnish Medical Society (MP), the Jane and Aatos Erkko Foundation (KH), the Finnish Cultural Foundation (MT, MP), the Sigrid Jusélius Foundation (KH), the Life and Health Medical Foundation (AS, MP), the Magnus Ehrnrooth Foundation (AS, KH), Finnish Society of Sciences and Letters (AS, KH), the Kone Foundation (AS), the Research Funds of University of Helsinki and Helsinki University Hospital (KH), Finska Läkaresällskapet (KH, MP), Fundação para a Ciência e a Tecnologia, I.P., under the Scientific Employment Stimulus - Institutional Call - CI-CTTI-94-ARH/2019 (DP).

## Conflict of Interest

The authors declare that the research was conducted in the absence of any commercial or financial relationships that could be construed as a potential conflict of interest.
