# Supplementary material for: The Human Bone Marrow Is Host to the DNAs of Several Viruses
Source: Front Cell Infect Microbiol. 2021 Apr 22;11:657245. doi: 10.3389/fcimb.2021.657245 (PMC8100435; doi:10.3389/fcimb.2021.657245)
Supplement: Supplementary file 1 [file DataSheet_1.pdf]

Supplementary Material

1 Supplementary Tables

Supplementary Table 1. In-house qPCR oligos and commercial qPCR assays used in this study.

**In-house assays**				
**qPCR**	**Oligo name**	**Target sequence (5'-3')**	**Organism**	**Reference**
Pan-B19V qPCR	B19MT1	CCACTATGAAAACTGGGCAATA	B19V	Toppinen *et al.* J Virol Methods 2015
	B19MT2	GCTGCTTTCACTGAGTTCTTCA		
	B19MTP1	FAM-AATGCAGATGCCCTCCACCCAG-BHQ1		
TTV-qPCR	AMTS	GTGCCGNAGGTGAGTTTA	TTV	Toppinen *et al.* FSIG 2020
	AMTAS	GGACTGGCCGGGCT		
	AMTAS gr4	GGTCTGGCCGGGCT		
	AMTPTU P	FAM-TCAAGGGGCAATTCGGGCT-BHQ1		
HERQ-9	HSV-1FWDLP1	GTTGAGCTAGCCAGCGA	HSV1	Pyöriä *et al.* mSphere 2020
	HSV-1REVLP1	CGTTAAGGACCTTGGTGAGC		
	HSV-1ProbeLP1	FAM-CGCGAACTGACGAGCTTTGTG-BHQ1		
	HSV2 FWD-2-2	CACACCACACGACAACAA	HSV2	
	HSV-2REVLP1	TAGTTCAAACACGGAAGCC		
	HSV-2probeLP1	JOE-CGGCGATGACGGCAATAAA-BHQ1		
	VZVFWDLP1	GCGCAAGGCTATTAGAGC	HHV3	
	VZVREVLP1	ACATGGCAGAAATCCCTG		
	VZVprobeLP1	TxRd-CGCATACCCGGAAGTTCTTCAGAT-BHQ2		
	H5 FWD211	GTGYTCCGTGAATCGTTAC	HHV5	
	H5 rev 211	AGTCKACCTCGATATCACAAGTCG		
	H5 Probe 20	TxRd-ACCCTGCTGCCGCCAGT-BHQ2		
	HHV6A FWD1-3	CGGCCTCCAGAGTTGTAA	HHV6A	
	HHV6A REV 10	TGTCCCTTCAACTACTGAATC		
	HHV6A Probe A1^	FAM-AC[+A]T[+G]TTGC[+T]A[+G]AAA[+G][+A]CT-BHQ1		
	HHV6A Probe A2^	FAM-AC[+A]T[+G]TTGC[+T]A[+C]AAA[+G][+A]CT-BHQ1		
	HHV6b FOTY1	TTTGACAGGAGTTGCTGAG	HHV6B	
	H6B ROTY 1	GGATTCAGGAAAAAGGTTCTAA		
	H6B PROBE MVP	JOE-AGGAAGCGTTTCGGTACACTTGGAG-BHQ1		
	HHV7 1. FWD	CTCGCAGATTGCTTGTTG	HHV7	
	HHV7 1. REV	GCATACACCAACCCTACTGTAA		
	H7 MOP PROBE	TxRd-TTAGGCATCACGTTGGCATTG-BHQ2		
	HHV8 fwd 3.1	ATATACGGCGACACTGACTC	HHV8	
	HHV8 REV 10	GAGCAGAAGGCACTTGAAG		
	H8 Probe 300	JOE-CGGAGGAGCTAGCGTCAATCA-BHQ1		

Supplementary Table 1 continues.

**qPCR**	**Oligo name**	**Target sequence (5'-3')**	**Organism**	**Reference**
MCPyV LT-qPCR	MCPyV-LT-F	CCACAGCCAGAGCTCTTCCT	MCPyV	Goh *et al.* EID 2009
	MCPyV-LT-R	TGGTGGTCTCCTCTCTGCTACTG		
	MCPyV-LT-P	FAM-TCCTTCTCAGCGTCCCAGGCTTCA-TAMRA		
RNaseP-qPCR	RnaseP-FWD	GAGGGAAGCTCATCAGTGGGG	human (RnaseP gene)	Toppinen *et al.* J Virol Methods 2015
	RnaseP-REV	CTTGGGAAGGTCTGAGACTAGGG		
	RnaseP-P	FAM-AGTGCGTCCTGTCACTCCACTC-BHQ1		
**Commercial assays used**				
**product code**		**assay name**	**Organims**	**Manufacturer**
HBV/ISEX/025		Hepatitis B Virus (HBV) PCR Kit, target: conservative sequence of open reading frame X (ORFx)	Hepatitis B virus	GeneProof, Genesig
Z-Path-HPV31-std		Human papillomavirus 31 Standard kit, target: E6 gene	Human papilloma-virus 31	PrimerDesign

^ Nucleotides in [ ] refer to locked nucleic acids

Supplementary Table 2. Viral reads of individual 4 that mapped to Merkel cell polyomavirus (Genbank accession number KF266964.1) by NCBI BLAST. The coverage profile is shown in Figure 3.

**read count**	**read sequence (5'-3')**	**BLAST**	
		**query coverage %**	**similarity %**
1	TATAATGATGGAATTGAACACCCTTTGGAGCAAATTCCAGCAAAATATCCACAAGCTCAGAAGTGACTTCTCTATGTTTGATGAGGGCAGTACAAAATTTCCT	100	98.06
1	GACTGGTGGCAAAAAACTTGAGAAGAAACTGACTACTGCTTACTGCCTCTGCACCTTTTCTCGACTCCTACTTCCTTCCTCTGTAAGTATTAGATATGGAAAAGTCTATAAGGCAAAATATCAAAGAAAGGTTATTTATGACAGATTTTCT	100	96.03
1	CCAACAAGCCGCAGACACTGTTCTAGCCACAAGGAGGTTAGA	100	95.24
1	ACCTTTTGAACTAGGCTGTGCTTTGGATAAATTTATGGTTGTTTTTGCGGCTGTGAAAGGGCAAAATAGCCTAAATA	100	96.10
1	CAGAACATGGAAATAAGAAAAAGAAGAATTCTTCAAAGTGGAACCACTGTATTGCTTTGTCTTATTTGGTGCTTGCCTGATACCACCTTTAAGCCTTGCTTACAAGAAGTAATTAAAAACTGGAAGCAAATTTTACAGAGTGAACTAGAGC	100	97.28
1	TCAAAGTGGAACCACTTTATTGCTTTGTCTTATTTGGTGCTTGCCTGATACAACCTTTAAGCCTTGCTTACAAGAAGAAATTAAAAACTGGAAGCAAA	100	100
1	GGCAGCAAATTTTACAGCGTGAAATATCAGATGGTAAATTTTGTCAAATGAGAGAAAATGTAGAAGCTGGTCAGGACCCTCTG	100	95.18
1	GTAAACAGTTTTCTCCTGCCCAAATTTATCTAAAAATCTGACAATATCAGGATCACCAGGTAATTGTTCTGCCCCCTCATATATACTGACCTCTTCTACCTGAGTATCTTTTCCTACCATAGGTTGGCCTGACACTTTTGGCATTAAGTTG	100	96.03
1	CCTCAATTTGGGTAATGCTATCTTCTCCAGTAACCACAGATAACAATTCCACTCGTCCTTTAACCAGCAGTTTTG	100	91.89
1	AATATCTTGCCCCACAGAATGCAGCAAGCTATTTTCCCACTGCAGAGGATCTAGGCTAAAGGCCATAAGTGCATGCCTCAAAACCTCATTACTACCTACCCACGAAACATCCCTCTTTACAAGTGAC	100	100
1	CACAGAATGCAGCAAGCTATTTTCCCACTGCAGAGGATCTAGGCTAAAGGCCATACGTGCATGCCTC	100	98.51

Supplementary Table 3. Viral reads of individual 21 that mapped to Torque teno virus (Genbank accession number AF122914.3) by NCBI BLAST. The coverage profile is shown in Figure 3.

read count	read sequence (5'-3')	BLAST	
		query coverage %	similarity %
1	AATGGCTGAGTTTTCCACGCCCGTCCGCAGCGGTGAAGCCACGGAGGGAGCTCAGCGCGTCCCGAGGGCGGGTGCCGGAGGTGAGTTTACACACCGCAGTCAAGGGGCAATTCGGGCTCGGGA	100	100
1	CGGCAGAGGATCCGCGCGTCCCGAGGGCGGGTGCCGAAGGTGAGTTTACACACCGAAGTCAAGGGGCAATTCGGGCTCGGGACTGGCCGGGCTATGGGCAAGACTCTTAAAA	91	95.10
1	GGACATCTACCGCGTCCCGAGGGCGGGTGCCGAAGGTGAGTTTACACACCGGAGTCAAGGGGCAATTCGGGCTCGGGACTGGCCGGGCTATGGGCAAGGCTCTTAAGGTTA	86	96.88
1	CGAGCGTCCCGAGGGCGGGTGCCGGAGGTGAGTTTACACACCGCAGTCAAGGGGCAATTCGGGCTCGGGACTGGCCGGGCCATGGGCAAGACTCTTAAAAAG	96	96.94
1	AGGGCGGGTGCCGTAGGTGAGTTTACACACCGGAGTCAAGGGGCAATTCGGGCTCGGGACTGGCCGGACCCCGGGCAA	100	92.31
1	CGAAGGTGAGTTTACACACCGGAGTCAAGGGGCAATTCGGGCTCGGGACTGGCCGGGCTATGGGCAAGGCTCTTAAAAAAGCATGTTTCTTGGTAGGGTACACCGAAAGAAAA	100	91.23
1	GAGGTGAGTTTACACACCGCAGTCAAGGGGCAATTCGGGCTCGGGACTGGCCGGGCTATGGGCAAGGCTCTTAAAAAGGCATGTTTCTCGGTAAACTCTACA	84	96.55
1	GAGTCAAGGGGCAATTCGGGCTCGGGACTGGCCGGACCCCGGGCAAGGCTCTTAAAAACGCCATGTTTT	97	91.04
1	CAGTCAAGGGGCAATTCGGGCTCGGGACTGGCCGGGCTATGGGCAAG	100	97.87
1	CAAGGGGCAATTCGGGCTCGGGACTGGCCGGGCTATGGGCAAGGCTCTTAAAAAGGCA	98	96.49
2	GAAAAGAAAAGGCAGACGTAGAAGAAAAGCAAAAATAATAATAAGACAGTGGCAGCCAAACTACAGAAGAAGATGTAATATAGTAGGCTACCTTCCTATACTTATCTGTGGTGAAAATACTGTTTCTAGAAACTATGCCACACACTCAGAC	100	97.35
1	GTGGCAGCCAAACTACAGAAGAAGATGTAATATAGTAGGCTACCTTCCTATACTTATCTGTGGTGAAAATACTGTTTCTAGAAACTATGCCA	100	96.74

Supplementary Table 2 continues.

read count	read sequence (5'-3')	BLAST	
		query coverage %	similarity %
1	CCTCTGTGACATGACACTACTAACTATCTATGCAACCGCAGCGGATATGCAATATCCGTTCGGCTCACCACTAACTGACACTGTGGTTGTTAACTTCCAGGTTCTGCAATCCATGTATGACAATACAATTAGCATATTACCTGAGGAAAAG	99	95.33
1	TATGCAACCGCAGCGGATATGCAATATCCGTTCGGCTCACCACTAACTGACACTGTGGTTGTTAACTTCCAGGTTCTGCAATCCATGTATGACAATACAATTAGCATATTACCTGAGGAAAAGTCAAACAGAGACAATCTTCTAACATCTA	100	89.40
1	TAGTACACACAAACCCCAAAAAAGGCTTTGTACCTTATTCTTTAAACTTTGGAAATGGTAAAATGCCAGGA	100	94.37
1	ACTTTGGAAATGGTAAAATGCCAGGAGGTAGTAGCAACGTTCCCATAAGAATGAGAGCTAAATGGTACCCCACTCTATTTCACCAACAAGAAGTTCTAGAGGCCCTAGCACAGTCAGGACCCTTTGCTTATCACTCAG	100	94.93
1	GCTGACACGCTCGCGCGTGTCAGACCACTTCGGGCTCGCGGGGGTCGGGAATTTTGCTAAACAGACTCCCAGTTGCCATTG	100	96.30
1	GCTGTGAATCAGTAACGAAAGTGAGTGGGGCCAGACTTCGCCATAAGGCCTTTAACTTTGGGTCGTCTGTCA	80	96.55
1	GGGGGACGGAATCTTTATTAAACAGACTCCAGGTTGCCATTGGACACTGGAGCTGTGAATTAGTAACGAAAGTGAGTGGGGCCAGACTTCGCCATAAGGCCTTTATCTTCTTGCCATTTGTCCGTGTCGGGGGTTGCCATAGGCTTCGGGC	100	88.08
1	TCTTTATTAAACAGACTCCAGGTTGCCATTGGACACTGGAGCTGTGAATTAGTAACGAAAGTGAGTGGGGCCAGACTTCGCCATAAGGCCTTTATCTTCTTGCCATTTGTCCGTGTCGGGGGTTGCCATAGGCTTCGGGCTCGGTTTTAGG	95	90.28

Supplementary Table 4. Viral reads of individual 25 that yielded by NCBI BLAST closest match (given as query coverage and similarity percentages) to human papillomavirus type 31 (Genbank accession number LR862018.1).

**read count**	**read sequence (5'-3')**	**BLAST**	
		**query coverage %**	**similarity %**
2	GAAAATGAAGACAGTAGTGATACTGGGGAGGATATGGTTGACTTTATTGACA<br>AT	100	100
2	GTCCCAAATGGTACAATGGGCATATGACAATGATGTTATGGATGATAGTGAA<br>ATTGCCTA	100	100
1	ACAAAGTTAGTGACGAAGGTGACTGGAGGGACATAGTAAAGTTTTTAAGATA<br>TCAACAAATAGAATTTGTGTCATTTTTATCTGCATTAAAGCTG	100	100
1	TTACATCTAATATAAATGCAGGTAAGGATGACAGATGGCCATACCTACATAGC<br>AGACTGGTGGTTTTTACATT	100	100
1	CACCTGTAGAAACATCAGGTCATTTACTACTTTCATCATCATCTATTAGCACAC<br>ATAATTATGAGGAAATACCTATGGATACATTTATTGTTTCTACTAATAATGAA<br>AACATAACAAGTAGCACACCCATTCCAGGGGTGCGCCGTCCTGC	100	99.34
1	ATCATCTATTAGCACACATAATTATGAGGAAATACCTATGGATACATTTATTG<br>TTTCTACTAATAATGAAAACATAACAAGTAGCACACCCATTCCAGGGGTGCGC<br>CGTCCTGCACGTTTAGGGTTATATAGTAAGGCTACACAACAAGTA	100	100
2	TGGCCTGATGTACCTATAGAGCATGCACCTACACAGGTTTTCCCATTTCCTTTG<br>GCCCCTACAACGCCACAAGTGTCTATTTTTGTTGATGGGGGTGATTTTTATTTG<br>CACCCTAGTTATTATATGTTAAAACGTCGACGTAAACGTGTAT	99	98.67
1	AATGGTTGCTGAGCCATATGGCGATACATTATTTTTTTATTTACGTAGGGAAC<br>AAATGTTTGTAAGGCATTTTTTTAATAGATCAGGC	100	98.86
1	GAATTTTGGATTGACCACACCTCCCTCAGGTTCTTTGGAGGATACCTATAGGTT<br>TGTCACCTCACAGGCCATTACATGTCAAAAAACTGCCCCCCAAAGGCCCAAGG<br>AAGATCCATTTAAAGATTATGTATTTTGGG	100	97.81
2	ACACCTTGCCAACATATAATCCAGTCCAACTTTGCAATTATACTATGAATCAT<br>GTTTGTTTAAATACAACTGTAGTTCAACTATGTGTC	100	97.75

Supplementary Table 5. The reconstructed sequences of parvovirus B19 derived from the same individuals´ bone and bone marrow. Mutation position is given from bone sequence.

**Case number**	**Genotype**	**B19V sequence in bone**		**B19V sequence in bone marrow**		**Mutation**
		**Genbank accession number**	**% of genome recovered (breadth)**	**Genbank accession number**	**% of genome recovered (breadth)**	
1	2	MT410184	99.9 %	MT988397	91.9 %	identical in the reconstructed genomic areas
6	2	MT410190	85.9 %	MT988403	88.8 %	at the nucleotide position 4095 G in bone, A in bone marrow (VP1/2, synonymous)
8	2	MT410186	87.2 %	MT988398	75.8 %	at the nucleotide position 352 G in bone, T in bone marrow (noncoding region)
						at the nucleotide position 4276 C in bone, T in bone marrow (VP1/2, synonymous)
						at the nucleotide position 5235 C in bone, A in bone marrow (noncoding region)
						at the nucleotide position 5302 A in bone, C in bone marrow (noncoding region)
10	1	MT410187	100 %	MT988400	97.2 %	at the nucleotide position 2032 G in bone, A in bone marrow (NS1, synonymous)
						at the nucleotide position 2114 C in bone, T in bone marrow (NS1, synonymous)

NS1= non-structural protein 1; VP1/2= capsid protein ½

2 Supplementary File References

Goh, S., Lindau, C., Tiveljung-Lindell, A., and Allander, T. (2009). Merkel cell polyomavirus in respiratory tract secretions. *Emerging Infectious Diseases* 15, 489–91. doi:10.3201/eid1503.081206.

Pyöriä, L., Jokinen, M., Toppinen, M., Salminen, H., Vuorinen, T., Hukkanen, V., et al. (2020). HERQ-9 Is a New Multiplex PCR for Differentiation and Quantification of All Nine Human Herpesviruses. *mSphere* 5, e00265-20. doi:10.1128/mSphere.00265-20.

Toppinen, M., Norja, P., Aaltonen, L. M., Wessberg, S., Hedman, L., Söderlund-Venermo, M., et al. (2015). A new quantitative PCR for human parvovirus B19 genotypes. *Journal of Virological Methods* 218, 40–45. doi:S0166-0934(15)00082-8.

Toppinen, M., Pratas, D., Väisänen, E., Söderlund-Venermo, M., Hedman, K., Perdomo, M. F., et al. (2020). The landscape of persistent human DNA viruses in femoral bone. *Forensic Science International: Genetics* 48, 102353. doi:10.1016/j.fsigen.2020.102353.
